# A rare differential diagnosis to occupational neck pain: bilateral stylohyoid syndrome

**DOI:** 10.1186/1745-6673-1-14

**Published:** 2006-06-26

**Authors:** Gertrud Kirchhoff, Chlodwig Kirchhoff, Sonja Buhmann, Karl-Georg Kanz, Miriam Lenz, Tobias Vogel, Rainer Maria Kichhoff

**Affiliations:** 1 Institut für Arbeitsmedizin, Universitätsmedizin Charité der Freien Universität Berlin und der Humboldt Universität zu Berlin, Ostpreussendamm 111, 12207 Berlin, Deutschland; 2 Chirurgische Klinik und Poliklinik-Innenstadt der Ludwig-Maximilians Universität München, Nussbaumstrasse 20, 80336 München, Deutschland; 3 Institut für Klinische Radiologie, Maximilians-Universität, Klinikum der Universität München, Ludwig-Maximilians-Universität, Marchioninistraße 15, 81377 München, Deutschland

## Abstract

Chronic neck pain is widely prevalent and a common source of disability in the working-age population. Etiology of chronic neck pain includes neck sprain, mechanical or muscular neck pain, myofascial pain syndrome, postural neck pain as well as pain due to degenerative changes.

We report the case of a 42 year old secretary, complaining about a longer history of neck pain and limited movement of the cervical spine. Surprisingly, the adequate radiologic examination revealed a bilateral ossification of the stylohyoid ligament complex. Her symptoms remained intractable from conservative treatment consisting of anti-inflammatory medication as well as physical therapy. Hence the patient was admitted to surgical resection of the ossified stylohyoid ligament complex. Afterwards she was free of any complaints and went back to work.

Therefore, ossification of the stylohyoid ligament complex causing severe neck pain and movement disorder should be regarded as a rare differential diagnosis of occupational related neck pain.

## Background

Chronic neck pain is widely prevalent and a common source of disability in the working-age population. The clinical picture includes stiffness and/or pain in the dorsal cervical region between the occipital condyles and the vertebral prominence of C7 [1]. Several studies have shown significantly reduced range of cervical movement and therefore a high rate of work disability followed by yearly accumulating cost for the compensation by insurance carriers.

Many researchers have tried to classify neck pain and many different methods have been proposed. The best and most widely accepted method of classification for neck pain is diagnostic triage, where patients are categorized as falling into one of three groups: serious spinal pathology; neurological involvement; and non-specific neck pain.

In the less number of cases, neck pain is caused by tumours, systemic arthropathy (e.g. rheumatoid arthritis, ankylosing spondylitis), infectious diseases, disorders of the thyroid gland, oesophageal obstruction or reflux disease [2]. Yet, in approximately 95% of patients with neck pain account for category three presenting a benign diagnosis, for example neck sprain, mechanical or muscular neck pain, myofascial pain syndrome, postural neck pain, pain due to degenerative changes as well as large osteophytes [3,4].

Against this background, we present a highly interesting etiology for chronic neck pain, which might be underestimated in current research.

## Case report

A 42-year old woman presented at our occupational outpatient clinic with major cervical pain increasing over the last two years. Anamnesis revealed that the patient has been working as a secretary in an open-plan office for 22 years with spending the major working hours sitting at a monitor workstation. The woman reported about severe neck pain, especially increasing over the last few months. Finally her daily work was limited due to pain to a maximum of three hours daily. No traumatic insult of the cervical spine was recalled, as well as the patient performed no sportive activity especially stressing the cervical spine.

The physical examination revealed a discrete muscle rigidification in the area of the lateral cervical musculature. The range of motion in the cervical spine was significantly reduced concerning extension/flexion with 15°-0°-10°, right lateral/left lateral flexion with 20°-0°-20° as well as right/left rotation with 10°-0°-10°. However, neurological examination showed overall inconspicuous findings. Pain during the last week, assessed by the 100 mm visual analogue scale (range from 0 to 100, 0 means no pain or disability and 100 means maximal pain or disability) accounted for a mean of 70 ± 15 mm.

**Figure 1:**
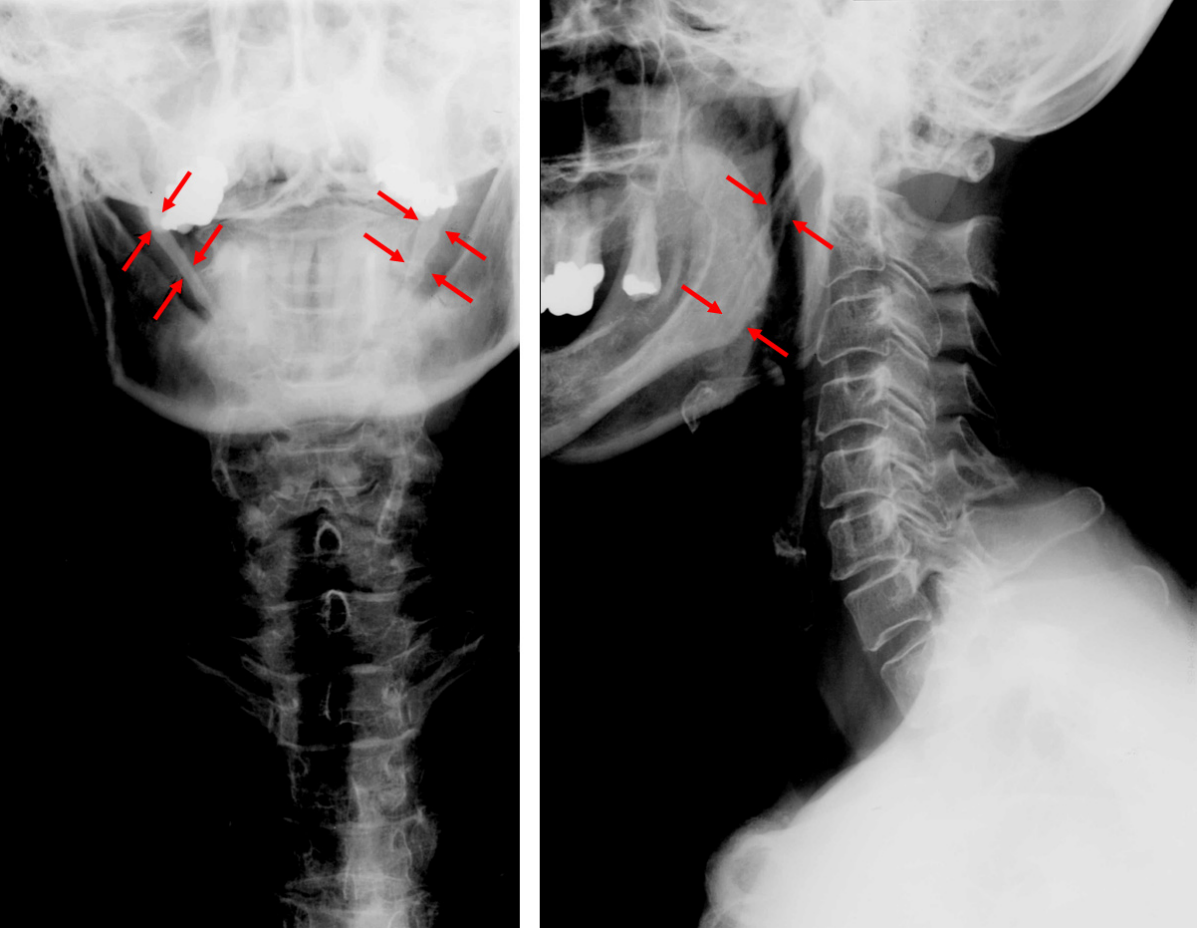
Radiographs of the vertebral spine: a-p and lateral view. Neither distinct malposition nor major degenerative changes of the cervical spine are recognizable. Formally and structurally inconspicuous cervical vertebral bodies and adnexa. But detection of a largely ossification of the ligamenta stylohyoidea on both sides. The patient's medical condition might be ascribed to a kerato-stylohyoidal syndrome.

**Figure 2:**
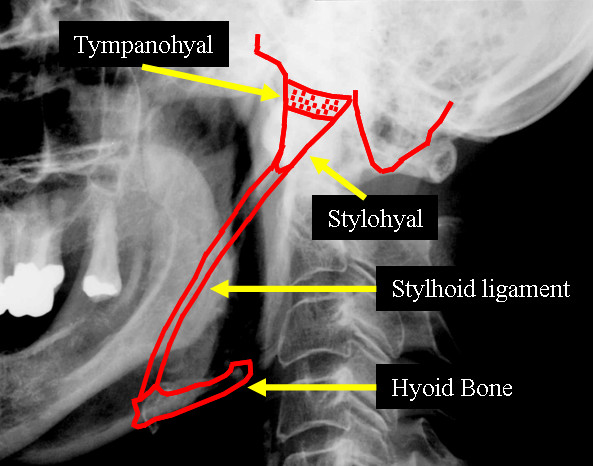
Schematic with anatomic situation at the styloid process and stylohyoid ligament.

Conventional a-p and lateral cervical spine radiographs did not reveal major pathological findings within the spine, but a complete bilateral ossification of the stylohyoid ligaments (Figure 1 and 2).

MRI as well as laboratory checks on inflammatory/rheumatoid processes revealed each normal finding.

In summary, no typical morphological correlatives for the patient's reproducible pain-symptoms and the limitation of movement of the cervical spine were found, except the bilateral ossification of the stylohyoid ligaments.

As the orthopaedic therapies, consisting of physical therapies as well as several courses of carbamazepine, baclofen, antidepressants and NSAID were ineffective the patient was finally presented to oral maxillofacial surgery for open resection of both ossified ligaments.

After the patient's dismissal from the hospital and an initial rehabilitation period, the patient now half a year later is free of pain. Movements of the head and neck improved significantly with a range of motion in extension/flexion of 45°-0°-40°, right lateral/left lateral flexion of 35°-0°-35° as well as right/left rotation of 20°-0°-20°. Pain during the last week, again assessed by the 100 mm VAS accounted for a mean of15 ± 5 mm.

The patient is now working again without any restriction in terms of pain-induced stop of the working process.

## Discussion

We report the case of a 42 year old secretary, complaining about a longer history of neck pain and limited movement of the cervical spine. Surprisingly, the adequate radiologic examination revealed a bilateral ossification of the stylohyoid ligament complex. After surgical resection the patient was free of any complaints and went back to work.

Although no general accepted criteria for classification exist, pain in the area of the neck/shoulder girdle is known for a work-relation and for presenting an economic problem in occupational health. In Germany, according to official statistics of compensation claims of the year 1993, the number of disorders especially affecting the cervical spine accounted for 2584 of all occupational conditioned disabilities and formed 18% of all indications concerning occupational disorders. Disorders of the spine represent the greatest item in terms of statistics concerning incapacity for work, and the medical condition causing neck and shoulder pain leads to average sickness duration of 26.1 days, in total breeding a gross domestic product deficit of 7.1 billion Euro.

More than 60 years ago, it was first noted that the stylohyoid ligament can cause throat pain if it ossified, although this was originally only associated with prior regional trauma or surgery [5]. In 1989, Camarda et al. classified cervicopharyngeal pain due to stylohyoid calcification into 3 distinct entities. The first, most commonly known as Eagle's syndrome, requires the presence of recent neck surgery or trauma and clinical palpation of elongation of the styloid process-stylohyoid ligament complex, with no pretraumatic or presurgical evidence of any such elongation or ossification. The second and most common entity, the stylohyoid syndrome, involves no prior trauma or surgery, but rather radiographic evidence of elongation or ossification at a young age. In spite of the presence of ossification at an earlier age, these patients are generally older than 40 years at time of presentation. In the most common third entity, pseudostylohyoid syndrome, the patient describes the same classic symptoms but has no evidence of any elongation of ossification [6,7].

As our patient denied any history of traumatic injury or surgery but revealed significant radiographic evidence of ossification it is likely that her ossification was present from her childhood on. Although the reported incidence of radiographic stylohyoid ossification accounts for up to 28%, in most cases the pathology is only recognized when patients become symptomatic and undergo repetitive radiologic examinations.

The most common symptom of an ossified stylohyoid ligament is pharyngeal, submandibular, or ear pain, especially during yawning or head movements. Only in the less number of cases severe neck pain with limited cervical range of movement is reported [8,9].

Only severely symptomatic cases, which do not respond to physical therapy and anti-inflammatory medications, will require surgery [10,11].

## Summary

In patients suffering from cervico-brachial pain, mainly occurring during working hours, especially at worksites such as workstations, primarily an organic genesis of the pain needs to be excluded. Besides mechanical, muscular or myofascial pain and degenerative changes of the cervical spine, in rare cases diseases of the rheumatoid sphere, infectious or tumorous diseases may be possible ethiologies. In terms of a very rare occurring entity, calcification and/or ossification of the stylohyoid ligament on one or both sides as described for the presented case, can lead to a similar complex of symptoms.

Concerning diagnosis and treatment of we recommend a close collaboration with orthopaedic surgeons and radiologists. Concerning diagnosis of cervical pain disorders an intensive clinical examination including occupational anamnesis is required. Adequate radiological evaluation of the cervical spine will be of essential character for the diagnostic process. Concerning chronic cervical pain caused by the stylohyoid syndrome the primary treatment is conservative pain therapy, including pain medication as well as physical therapy. In respect to therapy resistant symptoms surgical resection has to be considered.
